# Early diagnosis of type 2 diabetes based on multiple biomarkers and non-invasive indices

**DOI:** 10.3164/jcbn.17-81

**Published:** 2017-12-27

**Authors:** Aya Umeno, Toshiki Fukui, Yoshiko Hashimoto, Masatoshi Kataoka, Yoshihisa Hagihara, Hidenori Nagai, Masanori Horie, Mototada Shichiri, Kohzoh Yoshino, Yasukazu Yoshida

**Affiliations:** 1 Health Research Institute, National Institute of Advanced Industrial Science and Technology, 2217-14 Hayashi-cho, Takamatsu, Kagawa 761-0395, Japan; 2 Olive Takamatsu Medical Clinic. 649-8 Kankou-cho, Takamatsu, Kagawa 760-0076, Japan; 3 Biomedical Research Institute, National Institute of Advanced Industrial Science and Technology, 1-8-31 Midorigaoka. Ikeda, Osaka 563-8577, Japan; 4 School of Science and Technology, Kwansei Gakuin University, 2-1 Shigakuen, Sanda, Hyogo 669-1337, Japan

**Keywords:** diabetes, early detection, impaired glucose tolerance, oxidative stress, biomarker

## Abstract

We previously reported that type 2 diabetes risk, early impaired glucose tolerance and insulin resistance can be predicted by measuring the fasting levels of certain biomarkers. Here we validated these findings in randomly recruited healthy volunteers (*n* = 101) based on biomarker expression as well as various non-invasive indices. Weight, body mass index, waist circumference and visceral fat differed between individuals with impaired fasting glucose and/or impaired glucose tolerance, and normal subjects. Fasting plasma levels of glycated hemoglobin, leptin, pro-insulin and retinol binding protein 4 differed between impaired fasting glucose/impaired glucose tolerance and normal subjects group and between newly detected diabetes and normal subjects group. Insulin resistance was correlated with fasting levels of insulin and leptin/adiponectin (*r* = 0.913); of insulin, retinol binding protein 4 and leptin/adiponectin (*r* = 0.903); and of insulin, glycated albumin, and leptin/adiponectin (*r* = 0.913). Type 2 diabetes risk, early impaired glucose tolerance and insulin resistance were predicted with >98% specificity and sensitivity by comparing fasting glucose levels to the estimated Matsuda Index based on fasting levels of insulin, adiponectin and leptin with or without oxidative lineolate metabolites. Non-invasive indices are slightly correlated with glucose tolerance and insulin resistance but do not increase the accuracy of predicting type 2 diabetes risk.

## Introduction

Early detection and treatment of isolated impaired fasting glucose (IFG), impaired glucose tolerance (IGT) and type 2 diabetes (T2D) can delay or prevent serious complications associated with T2D such as blindness, amputation, and renal disease. IFG and IGT are pre-diabetic states since they represent intermediate stages during the transition from normal glucose tolerance to diabetes,^(1)^ and are defined based on the following oral glucose-tolerance test (OGTT) results: IFG is a fasting plasma glucose (FPG) level of 100–125 mg/dl and 120-min PG after OGTT <140 mg/dl; and IGT is an FPG <100 and PG of 140–199 mg/dl. One study reported that an elevated 120-min PG concentration was associated with a decrease in whole-body, insulin-mediated glucose disposal.^(2)^

The Japan Diabetic Society classifies subjects with FPG values of 100–109 mg/dl as having “high-normal” (HN) glucose metabolism and recommend a 75-g OGTT to determine whether they are normal, borderline, or diabetic.^(3)^ Identifying and treating borderline (IFG, IGT and high normal) individuals is essential for diabetes prevention.

The pathogenesis of T2D mellitus is well documented.^(4)^ Insulin resistance is characterized by increased glucose uptake resulting from decreased peripheral muscle glucose uptake. Increased lipolysis and plasma free fatty acid levels stimulate glucose output, reduce peripheral glucose utilization, and impair β-cell function. Compensatory insulin secretion by pancreatic β-cells may initially maintain normal PG levels, but β-cell function is already abnormal at this stage; concomitantly, there is inappropriate glucagon release from pancreatic β-cells. Glucagon-like peptide-1 and glucose-dependent insulinotropic polypeptide (incretin) enhance β-cell insulin secretion and are therefore therapeutic targets for T2D treatment.^(4)^ The effects of the so-called ominous octet (increased lipolysis, glucose reabsorption, hepatic glucose production in the liver and glucagon secretion; decreased glucose uptake, insulin secretion and incretin levels; and neurotransmitter dysfunction) lead to hyperglycemia in T2D.

Preventing β-cell dysfunction is critical for preventing T2D development and progression. Assessing *in vivo* insulin secretion by β-cells is complex since it is influenced by multiple factors including systemic insulin sensitivity, hepatic insulin extraction, plasma-free fatty acids, and glucolipid toxicity.^(5)^ Insulin resistance, which is also a risk factor for T2D, can be quantified by homeostasis model assessment of insulin resistance (HOMA-IR) as (FPG × fasting insulin level)/405, where a normal level is <1.6 and insulin resistance is >2.5 according to the Japan Diabetes Society;^(3)^ and by the Matsuda Index (MI), which is calculated based on plasma glucose and insulin levels during the OGTT.

MI3 points are measured as 10,000/[(FPG × fasting plasma insulin level) × (mean OGTT glucose level × mean OGTT insulin level)]^1/2^ at 0, 60 and 120 min after the start of the OGTT, where a normal level is >3.^(6)^ Although glucose tolerance and insulin homeostasis are important factors for evaluating diabetes risk and maintaining health, few people in Japan undergo the OGTT since it is a time-consuming, costly, and optional test. Furthermore, when the OGTT is performed, glucose levels 120 min after the test are occasionally measured in the absence of insulin data, resulting in a lack of information on insulin homeostasis.

We previously proposed an algorithm for predicting IFG, IGT and the HN state as well as insulin homeostasis abnormalities without the OGTT based on fasting plasma levels of insulin, leptin and adiponectin.^(7,8)^ The present study reports the results of a 5-year cohort study in which we added non-invasive indices and other fasting biomarkers to this algorithm in order to increase the accuracy of detection. We collected data from a randomly selected group of healthy volunteers who took part in the clinical survey and did not receive any specific diagnoses of T2D or other illnesses.

## Materials and Methods

### Materials

Lipid-peroxidation products such as 8-iso-prostaglandin F_2_α (isoP), isoP-d_4_, 5-hydroxyeicosa-6*E*,8*Z*,11*Z*,14*Z*-tetraenoic acid (5-HETE), 12-hydroxyeicosa-5*Z*,8*Z*,10*E*,14*Z*-tetraenoic acid (12-HETE), and 15- hydroxyeicosa-5*Z*,8*Z*,11*Z*,13*E*-tetraenoic acid (15-HETE) were obtained from Cayman Chemical Company (Ann Arbor, MI); 13-hydroxy-9*Z*, 11*E*-octadecadienoic acid (13-Z,E-HODE), 9-hydroxy-10*E*, 12*Z*-octadecadienoic acid (9-E,Z-HODE), 13-hydroxy-9*E*, 11*E*-octadecadienoic acid (13-E,E-HODE), 9-hydroxy-10*E*, 12*E*-octadecadienoic acid (9-E,E-HODE), 10-hydroxy-8*E*,12*Z*-octadecadienoic acid (10-Z,E-HODE), 12-hydroxy-9*Z*,13*E*-octadecadienoic acid (12-Z,E-HODE), and 13S-hydroxy-10*E*,12*Z*-octadecadienoic-9,10,12,13-d_4_ acid (13-HODE-d_4_) were from Larodan Fine Chemicals (Malmo, Sweden); and linoleic and arachidonic acid were from Sigma-Aldrich (St. Louis, MO). Other materials were of the highest commercially available grade.

### Subjects and sample processing

A total of 101 healthy male 40- to 55-year-old volunteers with no history of disease including diabetes were recruited for this study. A 75-g OGTT was performed for 120 min after >10 h of fasting, with blood collected every 60 min in tubes containing ethylenediamine tetraacetic acid disodium salt. Plasma and erythrocytes were separated immediately after collection by centrifugation at 1,500 × *g* for 10 min at 4°C.^(7)^ The plasma was frozen and stored at –80°C until analysis. The study protocol was approved by the institutional review boards of the National Institute of Advanced Industrial Science and Technology and Olive Takamatsu Medical Clinic, and was carried out in accordance with the ethical standards established in the 1964 Declaration of Helsinki and its later amendments. All subjects provided written, informed consent after being made fully aware of the study purpose.

### Analysis of oxidative stress markers and oxidative indices with the diacron-reactive oxygen metabolite (d-ROM) and biological antioxidant potential (BAP) tests

The levels of HODEs, HETEs and isoP were measured as previously described,^(7,9)^ with slight modifications. The parent molecules—i.e., linoleates (LAs) and arachidonates (AAs)—were detected using the same protocol. Briefly, 200 µl plasma were mixed with 300 µl saline, and 500 µl methanol containing the internal standards 8-iso-PGF_2_α-d_4_ (5 ng), 13-HODE-d_4_ (5 ng), and 100 µM butylated hydroxytoluene were added to the samples. This was followed by hydroperoxide reduction using an excess of triphenylphosphine and saponification with 1 M KOH in methanol. The mixture was acidified with 10% acetic acid in water and extracted with chloroform and ethyl acetate. An aliquot of the sample was analyzed by liquid chromatography/tandem mass spectrometry (LC-MS/MS) on a TSQ Quantum Access Max system (Thermo Fisher Scientific, Waltham, MA). The Dionex Ultimate 3000 system (Thermo Fisher Scientific) used for high-performance (HP) LC consisted of an HPG-3400 RS pump, WPS-3000 TPL RS Well Plate autosampler, and TCC-3000 RS column compartment equipped with a Hypersil Gold ODS column (3.0 µm, 100 × 2.1 mm; Thermo Fisher Scientific) set at 40°C. Elution was performed using a gradient of solvent A (2 mM ammonium acetate in water) and solvent B (methanol:acetonitrile = 5:95) at a flow rate of 0.2 ml/min. The initial gradient composition was 80% A and 20% B. The composition was changed to 79% A and 21% B for 10 min and then to 0% A and 100% B for 45 min. Electrospray ionization was performed at a needle voltage of 3.0 kV. Nitrogen was used as the sheath gas (50 psi) and auxiliary gas (10 units). The capillary was heated to 300°C, and the mass spectrometer was optimized for maximum sensitivity. A specific precursor-to-product ion transition was achieved by monitoring selected reactions after collision-induced dissociation in negative mode. Argon was used as the collision gas, and the collision cell pressure was 1.5 mTorr. The precursor, product ions, and collision energy were determined after MS/MS optimization as follows: *m/z* = 353.1 and 193.1 at 26 eV for 8-iso-PGF_2_α; *m/z* = 357.1 and 197 at 26 eV for 8-iso-PGF_2_α-d_4_; *m/z* = 295.0 and 195 at 18 eV for both 13-Z,E-HODE and 13-E,E-HODE; *m/z* = 295.0 and 171 at 18 eV for both 9-E,Z-HODE and 9-E,E-HODE; *m/z* = 295.0 and 183 at 18 eV for both 10-Z,E-HODE and 12-Z,E-HODE; *m/z* = 299.0 and 198 at 18 eV for 13-HODE-d_4_; *m/z* = 319.0 and 115 at 14 eV for 5-HETE; *m/z* = 319.0 and 163 at 14 eV for 12-HETE; *m/z* = 319.0 and 203 at 14 eV for 15-HETE; *m/z* = 279 at 5 eV for LA; and *m/z* = 303 and 259 at 12 eV for AA.

The d-ROM and BAP tests were performed using an automatic biochemical analyzer (Hitachi 7180; Hitachi, Ibaraki, Japan).^(10,11)^ d-ROM levels are expressed in Carratelli units.

### Detection of other biomarkers

Plasma levels of glycated hemoglobin (HbA1c), glucose, insulin, leptin, adiponectin, RBP4, glycated albumin, and high-sensitivity C-reactive protein (hs-CRP) were measured using the following commercially available enzyme-linked immunosorbent assay (ELISA) kits: HbA1c, RAPIDIA Auto HbA1c-L (Fujirebio, Tokyo, Japan); glucose, Cica Liquid GLU J (Kanto Chemical Co., Tokyo, Japan); insulin, Lumipulse Presto Insulin (Fujirebio, Tokyo, Japan); leptin, Human Leptin RIA kit (Millipore, Tokyo, Japan); adiponectin, CircuLex Human Adiponectin ELISA kit CY-8050 (MBL Co., Nagano, Japan); RBP4, CircuLex Human RBP4 ELISA kit (MBL Co.); glycated albumin, Lucia GA-L (Asahi KASEI Pharma Co., Tokyo, Japan); and hs-CRP, CircuLex Human HS-CRP ELISA kit CY-8071 (MBL Co.).

### Non-invasive indices

We measured several non-invasive indices including height, weight, waist circumference, diastolic blood pressure, systolic blood pressure, pulse-wave velocity, and abdominal fat distribution. The last two have been linked to early-stage diabetes.^(11)^ The branchial ankle pulse wave velocity of each subject was measured using an automatic oscillometer (PWV/ABI, BP-203RPE; Omron Collin, Tokyo, Japan) with subjects in the supine position after 5 min of bed rest. Abdominal fat distribution was determined by computed tomography with the subjects in the same position. Subcutaneous and intra-abdominal fat were measured at the level of the umbilicus by computed tomography.

### Statistical methods

Data are expressed as mean ± SD and were analyzed using IBM SPSS Statistics ver. 21.0. (IBM Corp, New York). One-way analysis of variance was used to evaluate the effect of clinical status on each index. Significant effects were further assessed by Tukey’s honestly significant difference multiple-comparisons test, and correlations were analyzed with the Pearson test. *P* values <0.05 were considered statistically significant.

## Results

### Subject characterization with the OGTT

**Fig. 1:**
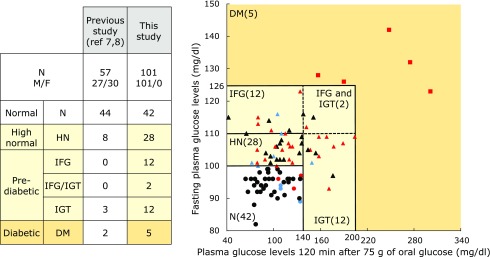
OGTT and classification of glucose tolerance using the OGTT. Circles, N (normal) group; triangles, “high-normal,” IFG and/or IGT groups; squares, diabetic group. Symbols in black, normal insulin resistance; blue, borderline insulin resistance; red, insulin resistance. Insulin resistance was determined using the HOMA-IR and MI3 indices.

The characteristics and metabolic parameters of subjects after the 75-g OGTT are shown in Fig. 1. Of the 101 male volunteers, 42 were characterized as normal (Group N); 28 were HN (fasting glucose levels of 100–109 mg/dl) without IGT (Group HN); 26 were IFG and/or IGT (Group IFG/IGT); and 5 were diabetic (Group D). The numbers of IFG and IGT patients were much higher than those previously reported.^(7,8)^

There were significant differences in weight, body-mass index (BMI), waist circumference, and visceral fat between Group IFG/IGT and Group N (Table 1). The levels of HbA1c, leptin, pro-insulin and RBP4 also differed between these two groups and between Groups D and N. There was no difference between Groups HN and N for these indices. However, in Group HN, RBP4/adiponectin and MI3 levels were lower whereas HOMA-IR was higher as compared to Group N.

HOMA-IR and MI3 have been proposed as indices for insulin resistance and homeostasis assessment although they rely on distinct criteria; the former is defined as a HOMA-IR index >2.5 and the latter as a MI <4.^(6,12)^ We defined borderline insulin resistance as values between 1.6 and 2.5. Based on HOMA-IR criteria and/or MI, in the present study 33 subjects were fully resistant and 10 were borderline resistant to insulin (Fig. 1 and Table 2). Three subjects in Group N and 11 in Group HN were diagnosed with abnormal insulin resistance. Additionally, 3 subjects in Group N and 4 in Group HN presented with borderline insulin resistance. Groups HN and IFG/IGT showed similar insulin resistance profiles, suggesting that a fasting glucose level >100 mg/dl is the threshold of insulin resistance.

### Oxidative stress status (d-ROM) and antioxidant capacity (BAP) in the clinical survey

We measured d-ROM and BAP levels as indicators of oxidative stress and antioxidant capacity, respectively. Both were determined photometrically as an increase in the absorption of dye released by the conversion of hydroperoxide to radicals (for d-ROM) and the production of Fe(II) from Fe(III) by antioxidants in the blood (for BAP). There were no changes in plasma levels of d-ROM or BAP among groups (Table 1). Furthermore, neither parameter was correlated with glucose levels or insulin resistance indices (not shown). On the other hand, BAP levels were positively correlated with the stereo-isomer ratio (ZE/EE) of HODEs, which reflects *in vivo* antioxidant capacity.^(9)^

### Correlation between glycometabolism markers (insulin, leptin and adiponectin) and insulin resistance index

**Fig. 2:**
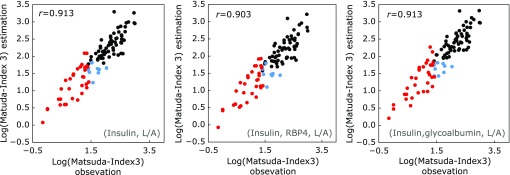
Estimation of MI3 by measurement of fasting plasma levels of biomarkers. A multiple linear-regression model was developed to predict MI3 based on eight physiological variables, including FPG and fasting levels of 10- and 12-Z,E-HODE/LA, HbA1c, RBP4, glycated albumin, leptin/adiponectin and hs-CRP. Correlations were observed between MI3 and (A) insulin, leptin and adiponectin; (B) insulin, RBP4, leptin and adiponectin; and (C) insulin, glycated albumin, leptin and adiponectin. Black, non-insulin resistance; blue, borderline insulin resistance; red, insulin resistance. Insulin resistance was determined based on HOMA-IR and MI3 indices.

 We previously reported that fasting levels of insulin, leptin, and adiponectin can be used to predict the risk of diabetes.^(8)^ In this study, insulin resistance was estimated based on PG and insulin levels during fasting and the OGTT. As previously reported,^(7,8)^ the MI3 index was more sensitive than the HOMA-IR index to insulin resistance, since the former is calculated based on glucose and insulin levels during the OGTT whereas the latter is calculated based on fasting glucose and insulin levels. Furthermore, HOMA-IR did not show a strong correlation when the glucose-clamp technique was used to measure fasting blood glucose levels >140 mg/dl or was used in non-obese subjects. MI3 determined by the OGTT was correlated with values obtained from fasting levels of insulin, leptin and adiponectin (Fig. 2A, *r* = 0.913); insulin, leptin, RBP4 and adiponectin (Fig. 2B, *r* = 0.903); and insulin, leptin, glycated albumin and adiponectin (Fig. 2C, *r* = 0.913), suggesting that the combination of insulin, leptin and adiponectin is sufficient for predicting insulin resistance.

### Detecting T2D risk using insulin, leptin/adiponectin, and 10- and 12-Z,E-HODE/LA

**Fig. 3:**
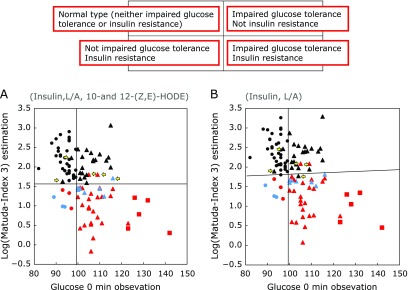
Predicting risk of glucose tolerance and insulin resistance. (A, B) MI3 was estimated by measuring insulin and leptin/adiponectin with (A) and without (B) 10- and 12-(Z,E)-hydroxyoctadecadienoic acid. Circles, N (normal) group; triangles, “high-normal,” IFG and/or IGT groups; squares, diabetic group. Symbols in black, normal insulin resistance; blue, borderline insulin resistance; red, insulin resistance. Insulin resistance was determined based on HOMA-IR and MI3 indices. The yellow arrow represents patients for whom we were unable predict the type of glucose tolerance and insulin resistance.

We previously developed an algorithm that uses fasting levels of 10- and 12-Z,E-HODE/LA, insulin, and leptin/adiponectin to assess the utility of biomarkers for detecting early-stage diabetes,^(7,8)^ and used it here to evaluate IGT and insulin resistance, including in borderline cases. The combination of 10- and 12-Z,E-HODE/LA, insulin, and leptin/adiponectin and insulin and leptin/adiponectin showed >98% specificity and sensitivity. The cutoff value for the y-axis was determined by adding data for 10- and 12-Z,E-HODE/LA (Fig. 3A).

### Application of non-invasive indices to the algorithm

**Fig. 4:**
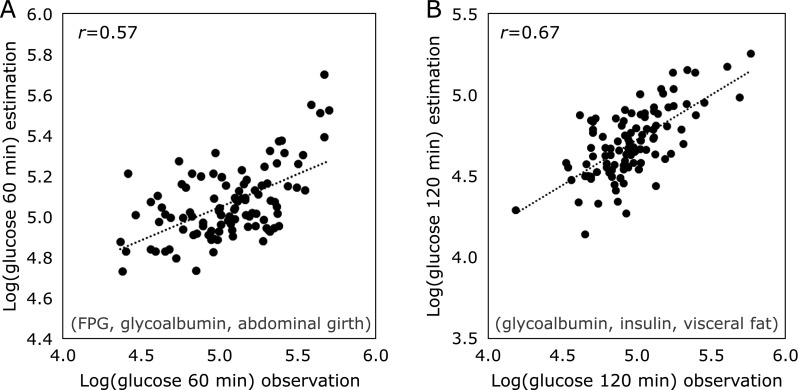
(A, B) Estimation of glucose levels (A) 60 min and (B) 120 min after the OGTT, based on fasting biomarker levels and non-invasive indices. Several variables including glucose, insulin, HbA1c, leptin, adiponectin, RBP4, glycoalbumin, waist circumference, pulse wave-propagation speed, systolic blood pressure, diastolic blood pressure, BMI and visceral fat were highly correlated with glucose levels (*p*<0.01) or with the MI3 (*p*<0.05) at both time points. Logarithmic transformations were applied to all variables to achieve normality prior to analysis. Stepwise variable-selection analysis was performed. The criterion for including or excluding variables in the model was based on F statistics, using a critical *p* value of 0.05. Fasting levels of glucose and glycoalbumin along with waist circumference (A), and fasting levels of glycoalbumin and insulin along with visceral fat (B) were analyzed.

We evaluated whether the inclusion of non-invasive indices could increase the precision of our algorithm. BMI, waist circumference, and visceral fat were correlated with fasting, 60 and 120 min PG in the OGTT and with MI3 and HOMA-IR (Table 3). The pulse wave-propagation speed was correlated only with MI3, whereas systolic and diastolic blood pressure was correlated with MI3, 120-min PG level after OGTT and HOMA-IR. We applied these indices to the algorithm by a stepwise variable-selection method to predict MI3 values and 60- and 120-min PG levels after OGTT; however, the non-invasive indices did not increase the precision of the algorithm for predicting MI3. In contrast, the combination of fasting levels of glucose and glycoalbumin and waist circumference could predict PG levels 60 min after OGTT (*r* = 0.57; Fig. 4A), while the combination of fasting levels of insulin and glycoalbumin and visceral fat could predict PG levels 120 min after OGTT (Fig. 4B, *r* = 0.67) as explanatory variables in the multiple linear regression model.

## Discussion

There were more borderline (IFG and/or IGT) subjects in the present study than in our earlier study (*n* = 26, 25.8% vs *n* = 3, 5.3%) (Fig. 1). We previously recruited similar numbers of male and female subjects (Tokushima in Shikoku Island), but in the present study (Kagawa in Shikoku Island), only male subjects were included in order to avoid physiological variations specific to females such as menstruation that could potentially confound the results. Indeed, it was reported that 4-hydroxy-2-nonenal levels (a measure of oxidative stress) differed significantly between healthy pre- and postmenopausal women subjected to the mental and physical stress of a word–color test.^(13)^

Japanese laws mandate annual health checks for workers; this includes measurement of FPG and HbA1c levels. However, these examinations lack the OGTT and IGT cannot be detected. When FPG value exceeds 110 mg/dl, subjects are usually advised to undergo a comprehensive health check including an optional OGTT. In this study, this recommendation was made to 19/101 subjects (12 subjects with IFG; two subjects with IFG and IGT; and five subjects with DM) (Fig. 1). Surprisingly, of the 38 subjects in the HN group (including 10 with IGT), 24 showed insulin resistance or were borderline. The Japan Diabetes Society has reported that approximately 25–40% of HN subjects develop pre-diabetes and diabetes. Importantly, nine subjects (FPG <100) with IGT (including two with IGT) or insulin resistance (including seven in Group N) were overlooked in the annual health check. Our multiple linear-regression model analyzing fasting glucose levels against 10- and 12-Z,E-HODE/LA, insulin and leptin/adiponectin or against insulin and leptin/adiponectin (Fig. 3) predicted IGT and insulin resistance without the OGTT (Fig. 1), with specificity and sensitivity >98%. Furthermore, six subjects showed abnormal insulin resistance with <99.5 mg FPG/dl.

The OGTT is a useful tool for identifying those at risk of glucose tolerance and insulin resistance, since IGT is largely due to the latter whereas IFG is due to dysregulated gluconeogenesis. However, the OGTT is a time-consuming and invasive test. Adiponectin and leptin are secreted exclusively by adipose tissue and act as hormones with antagonistic effects. The results of this study demonstrate that multiple markers including insulin, leptin/adiponectin, and 10- and 12-Z,E-HODE/LA can be used to detect diabetes risk at an early stage. Leptin has pro-inflammatory effects while adiponectin has insulin-sensitizing and anti-inflammatory properties. It is therefore reasonable that L/A is highly correlated with insulin levels during the OGTT. It is also interesting that measuring the levels of lipid oxidation metabolites (10- and 12-Z,E-HODE/LA) enabled us to set a cutoff value. The role of oxidative stress in the pathogenesis of T2D is still debated.^(7–9)^ Specific and reliable biomarkers are needed in order to clarify the pathogenic mechanism of T2D. We studied the oxidation products of linoleates because the formation of isomers can provide important mechanistic information: for instance, enzymatic and free radical-mediated oxidation yields four hydroperoxyoctadecadienoic acid (HPODE) isomers (namely, 9- and 13-Z,E-HPODE and 9- and 13-E,E-HPODE), while singlet oxygen oxidizes linoleates to generate 9- and 13-Z,E-HPODE and 10- and 12-Z,E-HPODE. HPODEs are readily reduced to HODEs *in vivo*. The 10- and 12-Z,E-HPODE examined in this study are specific oxidation products of singlet oxygen,^(7–9)^ which is produced *in vivo* by the reaction of hydrogen peroxide with hypochlorite derived from myeloperoxidase (Scheme 1). The latter is secreted by activated phagocytes^(14,15)^ or by eosinophils through a peroxidase-catalyzed mechanism.^(16)^ Singlet oxygen is also produced via bimolecular interactions between lipid peroxyl radicals (Scheme 2).^(17)^ However, the fact that fasting plasma levels of 9- and 13-Z,E-HODE and 10- and 12-Z,E-HODE were not correlated with glucose tolerance and insulin resistance in the OGTT (data not shown) makes it unlikely that singlet oxygen was formed via Scheme 2. Singlet oxygens may instead have been generated when neutrophils containing myeloperoxidase were recruited to adipose cells or β-cells as a result of hyperglycemia, which is considered as the early stage of diabetes pathogenesis and a normal response to inflammation before abnormal insulin secretion and insulin resistance are observed.^(18,19)^

Quantose M^Q^ consisting of insulin, α-hydroxybutyrate, linoleoylglycerophosphocholine, and oleate correlated well with insulin-stimulated glucose disposal in the ACT NOW clinical study.^(20)^ α-Hydroxybutyrate, linoleoylglycerophosphocholine, and oleic acid were useful biomarkers for identifying subjects with IGT independent of age, sex, BMI and fasting glucose without the need for the OGTT in a clinical study of European subjects without diabetes.^(21)^ However, these biomarkers are low-molecular weight compounds that were detected using expensive instrumentation, namely HPLC-MS/MS. In contrast, quantitative measurements and immunological assays for our biomarkers (i.e., insulin, adiponectin and leptin) are much easier to perform. Indeed, commercially available antibodies against insulin and leptin were reliable in the present study; we have also obtained an alpaca anti-adiponectin antibody that it highly stable against temperature fluctuations. Moreover, we recently developed a second-generation prototype of our multi-marker analysis system equipped with a circular dichroism (CD)-type microfluidics device^(22)^ and an apparatus for measuring chemiluminescence. ELISA can be rapidly performed on microfluidics devices, facilitating rapid detection of numerous biomarkers in single, small-volume samples. We have designed a 5-year cohort study to evaluate multiple biomarkers with the abovementioned analysis system. Over 100 subjects have been recruited and will be administered the OGTT for 5 consecutive years. We believe that these multiple biomarkers will provide sufficient information to detect diabetes risk during annual health examinations. Lifestyle modification or pharmacotherapy with metformin plus low-dose pioglitazone has been recommended for patients with IFG ± IGT.^(23)^ It was also reported that chronic treatment with partially hydrolyzed guar gum (PHGG) improved insulin resistance, delayed the onset of diabetes, and inhibited the development of diabetic complications and that cysteinylated transthyretin was identified as a predictive biomarker of treatment response to PHGG in Long-Evans Tokushima Otsuka rats.^(24)^ We expect our CD-type microfluidics device to provide information not only for predicting IGT and insulin resistance, but also on the effectiveness of drugs and dietary changes.

Non-invasive indices did not perform well using our algorithm although they were individually linked to IGT and/or insulin resistance (Table 3). This is expected given that these indices depend on long-standing lifestyle choices. However, fasting plasma levels of insulin and glycoalbumin and visceral fat were correlated with fasting glucose levels 120 min after the OGTT (*r* = 0.67; Fig. 4B). We applied this calculated value derived from fasting plasma levels of insulin, glycoalbumin, and visceral fat to the x axis instead of FPG in Fig. 3, but it lacked sufficient predictive power (data not shown). This result indicates that FPG was more informative than the combination of insulin and glycoalbumin levels and visceral fat for predicting IGT and insulin resistance, although this requires confirmation in a cohort study.

The results presented here confirm the robustness and effectiveness of our proposed algorithm, which can predict diabetes risk, glucose tolerance, and insulin resistance before diabetes onset. At a minimum, the algorithm consisted of fasting plasma insulin and leptin/adiponectin levels, and preferably 10- and 12-Z,E-HODE levels. In addition, the OGTT was useful when insulin data were obtained. However, given that the OGTT is a time-consuming, invasive and inconvenient test that is limited to healthy individuals, we propose that it be replaced by a more convenient annual health examination that measures only the 4 fasting plasma biomarkers confirmed in this study, which are more reliable than plasma levels of HbA1c that reflect the accumulation of >3 months of physical conditioning.

Diabetes can have fatal complications such as diabetic nephropathy, myocardial infarcted diabetes, and glaucoma; disease detection at early stages is therefore critical.^(25)^ We suggest that this is possible using the set of biomarkers consisting of leptin, adiponectin, insulin, and 10- and 12-Z,E-HODE (as well as HbA1c), especially when used in conjunction with the CD-type microfluidic device. This can help to shift the focus of the medical community from T2D treatment to prevention.^(26,27)^

## Figures and Tables

**Table 1:** Subject characteristics at the time of the OGTT

		Group N	Group HN	Group IGT and/or IFG	Group D
		Normal type	High-normal type^d^	Pre-diabetic type	Diabetic type
*N*		42	28	26	5
Age (years)		48.0 ± 4.9	49.0 ± 4.2	49.6 ± 4.0	48.8 ± 4.7
Height (cm)		170.3 ± 6.7	170.3 ± 5.5	171.7 ± 5.6	172.3 ± 4.3
Weight (kg)		65.6 ± 10.3	69.8 ± 9.5	74.4 ± 14.0*	77.5 ± 8.3
BMI (kg/m^2^)		22.5 ± 2.8	24.0 ± 3.2	25.2 ± 4.8*	26.1 ± 2.9
SBP (mmHg)		119.8 ± 13.2	123.6 ± 12.5	126.1 ± 15	125.6 ± 11.7
DBP (mmHg)		76.6 ± 10.1	79.2 ± 10.1	80.7 ± 10.7	82.2 ± 5.9
Waist circumference (cm)		82.0 ± 7.4	84.5 ± 9.2	89.1 ± 11.1*	92.9 ± 6.1
d-ROM (CARR U)		265.4 ± 56.5	281.7 ± 59.2	281.7 ± 38.2	258.2 ± 27.4
BAP (µmol/L)		2,282.1 ± 202.1	2,242.4 ± 168.3	2,299.7 ± 202.8	2,242.2 ± 281.9
Visceral fat (cm^2^)		64.1 ± 37.2	76.2 ± 38.6	115.6 ± 57.7**	114.9 ± 35.7
PWV_R (cm/s)		1,370.4 ± 136.5	1,360.6 ± 192.6	1,418.7 ± 227.9	1,432.4 ± 177.9
PWV_L (cm/s)		1,359.5 ± 147.4	1,369.7 ± 208.6	1,406.5 ± 245.3	1,404.2 ± 192.5
HbA1c (%)		5.4 ± 0.3	5.5 ± 0.3	5.7 ± 0.4**	6.2 ± 0.3**
Glycoalbumin (%)		13.4 ± 1.2	13.1 ± 1.3	13.3 ± 1.5	14.5 ± 0.6
Leptin (ng/ml)		5.2 ± 2.6	7.1 ± 4.1	7.8 ± 4.3*	13.6 ± 7.9**
Adiponectin (µg/ml)		9.0 ± 4.2	7.3 ± 2.7	6.6 ± 2.8*	6.2 ± 1.1
pro-insulin (µU/ml )		7.3 ± 4.3	10.6 ± 6.4	15.6 ± 13.5**	33.4 ± 13.4**
RBP4 (mg/dl)		23.8 ± 6.1	24.9 ± 7.0	24.9 ± 5.5**	25.4 ± 3.0**
hs-CRP (mg/dl)		0.11 ± 0.20	0.16 ± 0.32	0.15 ± 0.25	0.13 ± 0.90

Glucose (mg/dl)	0^a^	94.4 ± 3.4	103.6 ± 2.7**	109.2 ± 6.4**	130.2 ± 7.4**
	60^b^	144.4 ± 37.6	149.0 ± 32.0	193.8 ± 43.7**	286.0 ± 12.0**
	120^c^	99.4 ± 19.6	105.1 ± 17.9	133.4 ± 40.2**	234.2 ± 59.6**
Insulin (µU/ml)	0	4.5 ± 2.3	7.3 ± 4.4	8.9 ± 7.5**	14.5 ± 5.5**
	60	56.8 ± 44.8	73.6 ± 45.6	97.1 ± 64.3**	95.3 ± 38.2
	120	33.8 ± 24.9	40.9 ± 26.6	85.6 ± 84.7**	165.2 ± 68.0**
HOMA-IR		1.0 ± 0.57	1.9 ± 1.1*	2.4 ± 2.0**	4.9 ± 1.9**
MI3		9.4 ± 4.5	6.3 ± 3.6*	5.1 ± 3.6**	1.7 ± 0.4**

Data are presented as mean ± SD. One-factor one-way analysis of variance was used to examine the main effects of the subject groups on each index. Significant effects were identified by Tukey’s honestly significant difference multiple-comparisons test. * *p*<0.05, ** *p*<0.01 vs Group N. ^a^Fasting, ^b^60 min after ingestion of 75 g glucose, ^c^120 min after ingestion of 75 g glucose, ^d^fasting sugar = 100–109 mg/dl. BAP, biological antioxidant potential; BMI, body-mass index; CARR U, Carratelli units; DBP, diastolic blood pressure; d-ROM, diacron-reactive oxygen metabolites; HOMA-IR, homeostasis model assessment of insulin resistance; hs-CRP, high sensitivity C-reactive protein; PWV, pulse-wave velocity; RBP4, retinol-binding protein 4; SBP, systolic blood pressure.

**Table 2:** Glucose tolerance and insulin resistance in the study subjects

	Group N			Group HN			Group IFG and/or IGT			Group D	
	Normal type			High-normal type			Pre-diabetic type			Diabetic type	
Total	42			28			26			5	
Insulin resistance											
None	36	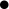		13	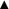		9	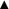		0	
	85.7%			46.4%			34.6%				
Borderline	3	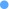		4	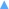		3	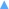		0	
	7.1%			14.3%			11.5%				
Abnormal	3	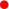		11	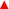		14	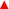		5	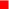
	7.1%			39.3%			53.8%				

Symbols in the table are as defined in Fig. 1 and 3.

**Table 3:** Correlations of non-invasive indices with glucose tolerance and insulin-resistance indices

	BMI	Abdominal girth	PWV (right)	PWV (left)	SBP	DBP	Visceral fat
HbA1c	(−)	(−)	(−)	(−)	(−)	(−)	(−)
FPG	**	**	(−)	(−)	(−)	(−)	**
60 min PG after OGTT	*	**	(−)	(−)	*	(−)	**
120 min PG after OGTT	**	**	(−)	(−)	*	*	**
MI3	^##^	^##^	^#^	^#^	^##^	^#^	^##^
HOMA-IR	**	**	(−)	(−)	**	*	**

*^,#^*p*<0.05, **^,##^*p*<0.01, (–) *p*>0.05 vs HbA1c and FPG in the OGTT, MI3 and HOMA-IR. * and ^#^ indicate positive and negative correlations, respectively. BMI, body-mass index; DBP, diastolic blood pressure; FPG, fasting plasma glucose; HOMA-IR, homeostasis model assessment of insulin resistance; PWV, pulse wave velocity; SBP, systolic blood pressure.
